# Neuronal Intranuclear Inclusion Disease: A Case Report With Insights Into Clinical Features and Potential Therapeutic Implications

**DOI:** 10.1155/crnm/3279987

**Published:** 2026-07-08

**Authors:** Lingqiong Xia, Jianhua Chen, Fuying Liu, Fei Feng, Nanqu Huang, Yong Luo

**Affiliations:** ^1^ Department of Neurology, Third Affiliated Hospital of Zunyi Medical University (The First People’s Hospital of Zunyi), Zunyi, Guizhou, China

**Keywords:** case report, early diagnosis, neurological symptoms, neuronal intranuclear inclusion disease, NIID

## Abstract

Neuronal intranuclear inclusion disease (NIID) is a chronic progressive neurodegenerative disorder characterized by the formation of eosinophilic intranuclear inclusion bodies in the nuclei of neurons in the central, peripheral, and autonomic nervous systems, as well as visceral organs. The clinical presentation of this disease is diverse, manifesting as headaches, fever, altered consciousness, dementia, seizures, sensory and motor disturbances, and autonomic dysfunction, making it prone to misdiagnosis in clinical practice. This article reports a case of NIID diagnosed through skin biopsy and genetic testing, along with a review of relevant literature. It provides an overview of the characteristics and diagnosis of NIID, emphasizing the importance of early diagnosis and comprehensive treatment in managing this rare neurological disorder.

## 1. Introduction

Neuronal intranuclear inclusion disease (NIID) is a chronic progressive neurodegenerative disorder characterized by the presence of eosinophilic intranuclear inclusions in the central and peripheral nervous systems, as well as visceral organs [1]. One of the imaging features includes high signal intensity along the gray–white matter junction on diffusion weighted imaging (DWI) in magnetic resonance imaging (MRI). The identification of nuclear inclusions through skin biopsy [2] and the discovery of pathogenic GGC repeat expansions in the NOTCH2NLC gene [3] can lead to the diagnosis of this disease. Clinical manifestations of NIID are diverse, including cognitive impairment, altered consciousness, behavioral abnormalities, stroke‐like episodes, autonomic dysfunction, peripheral neuropathy, movement disorders, and ataxia [4], with cognitive impairment being the most common symptom [5]. Here, we report a case of an NIID patient who presented with headaches accompanied by fever, and was ultimately diagnosed with NIID through skin biopsy and genetic testing. This case underscores the critical importance of early diagnosis and intervention in improving the prognosis of NIID.

## 2. Case Presentation

A 62‐year‐old female patient was admitted to our hospital with a chief complaint of “headache and fever for 1 day”. The patient developed a headache in January 2021 after exposure to cold, characterized by continuous throbbing pain in the frontal and bilateral temporal regions, accompanied by fever. At an outside facility, her temperature was measured at 37.5°C. She had no vomiting, altered consciousness, behavioral abnormalities, speech, or motor dysfunction. Following unsatisfactory treatment at a local hospital, she sought treatment at our hospital. The patient had a history of hypertension for 3 years. Three months prior, she experienced a decline in memory characterized by forgetfulness and an inability to recall past events. Additionally, she had intermittent gastrointestinal symptoms such as diarrhea and melena for the past 2+ months.

Upon admission, the patient had a body temperature of 37.3°C, respiratory rate of 20 breaths per minute, heart rate of 64 beats per minute, and blood pressure of 130/78 mmHg. The abdomen was soft, with slight tenderness in the lower abdomen, no rebound tenderness or muscle tension, and no abnormalities in the heart and lungs. Neurological examination: the patient was alert and oriented, with fluent speech, mild memory impairment, and normal higher mental functions. Pupils were equal and reactive to light, approximately 3 mm in diameter, with no eye movement disorders or nystagmus. Muscle strength was graded as 5/5 in all four limbs, normal muscle tone, normal tendon reflexes, intact deep and superficial sensations, no pathological signs elicited, normal coordination, and negative meningeal signs.

Laboratory tests: Complete blood count: white blood cells 10.6 × 10^9^/L, neutrophil ratio 88.8%, no other abnormalities noted. High‐sensitivity C‐reactive protein, liver and kidney function tests, lipid profile, fasting blood glucose, electrolytes, homocysteine, rheumatoid factor, and thyroid function tests were all normal. Hepatitis B surface antigen, HIV antibodies, and syphilis antibodies were negative. Colonoscopy showed no abnormalities. A chest computed tomography (CT) scan revealed localized atelectasis in the lower lobes of lungs, cardiomegaly, and bilateral pleural thickening. Electroencephalography (EEG) and brain topography: The background brain wave activity was predominantly composed of low‐to‐medium amplitude *α* waves at 8–10 Hz (20–40 μV), with poor modulation and poor morphology. *α* wave inhibition during visual response was suboptimal. Excess low‐to‐medium amplitude *θ* waves at 4–7 Hz were observed in all leads, along with an appropriate amount of low‐amplitude *β* waves at 14–24 Hz mixed in. The cranial CT scan (Figure 1A) indicated mild signs of cerebral atrophy, with decreased density around the bilateral lateral ventricles, centrum semiovale, and corona radiata, suggesting possible demyelinating lesions, and recommended an MRI examination. The cranial MRI revealed symmetric patchy low signal on T1‐weighted imaging (T1WI) and high signal on T2‐weighted imaging (T2WI) adjacent to the bilateral lateral ventricles, with high signal on fluid‐attenuated inversion recovery (FLAIR) sequence (Figure 1C), and high signal at the gray–white matter junction on DWI (Figure 1B). The apparent diffusion coefficient (ADC) sequence showed slightly decreased signal, and magnetic resonance angiography (MRA) (Figure 1D) revealed no abnormalities. Contrast‐enhanced cranial MRI (Figure 1E) showed no significant enhancement in the symmetric patchy long T1 and long T2 signal lesions adjacent to the bilateral lateral ventricles and corpus callosum.

**FIGURE 1:**
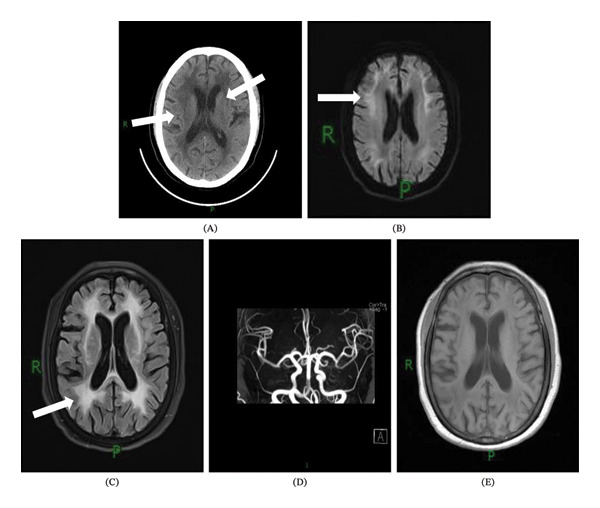
Imaging findings of the patient. (A) Head CT shows slightly hypodense areas around the bilateral ventricles, semioval centers, and corona radiata (indicated by the white arrows), suggesting possible demyelinating changes. (B) Cranial MRI DWI sequence reveals high signal intensity at the gray–white matter junction (indicated by the white arrows). (C) Head MRI FLAIR sequence shows high signal intensity at the gray–white matter junction (indicated by the white arrows). (D) Head MRA results show no significant abnormalities. (E) Head contrast‐enhanced MRI shows no abnormal signals.

On the third day after admission, the patient underwent the first lumbar puncture to measure the cerebrospinal fluid (CSF) pressure, which was 172 mmH_2_O. The CSF analysis showed abnormalities, with a protein level of 0.91 g/L (normal range 0.15–0.45 g/L), and no other abnormalities were noted. India ink staining and acid‐fast staining of the CSF were unremarkable. Initially suspected viral encephalitis, the patient was treated with acyclovir, leading to slight symptom improvement.

On the 11th day after admission, the patient underwent the second lumbar puncture to measure the CSF pressure, which was 130 mmH_2_O. The CSF analysis showed abnormalities, with a protein level of 0.80 g/L (normal range 0.15–0.45 g/L), and no other abnormalities were noted. India ink staining and acid‐fast staining of the CSF were unremarkable.

Due to the high signal at the cortical‐subcortical junction of the cerebral hemisphere on the patient’s cranial MRI DWI sequence, we conducted skin pathology and blood NOTCH2NLC gene testing. On the 19th day after admission, the results of the skin and muscle pathology report, as well as the genetic report, were returned: The skin and muscle pathology report revealed eosinophilic inclusion‐like structures in the sweat gland ductal epithelial cells, consistent with the characteristic changes of NIID in skin pathology (Figure 2A‐B). The genetic report (Figure 2C‐D) indicated that the patient’s blood sample, after undergoing TP‐PCR testing for the NOTCH2NLC gene, showed GGC repeat expansions with > 100 repeats in the 5′UTR region, suggesting a positive pathogenic gene for NIID. The final diagnosis was NIID.

**FIGURE 2:**
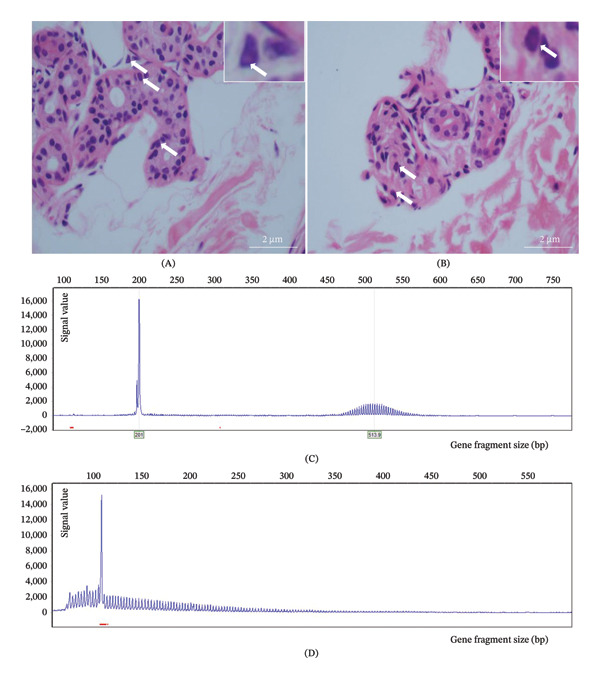
Pathological and genetic testing results. (A‐B) Skin pathology reveals eosinophilic inclusion‐like structures in the sweat gland ductal epithelial cells (indicated by the white arrows). (C‐D) Genetic testing of blood samples demonstrates a GGC repeat expansion exceeding 100 repeats in the 5′UTR region.

During the hospitalization, the patient presented with headache symptoms upon admission, and relevant examinations were not fully completed at that time. Therefore, symptomatic treatment with carnosine was administered to improve brain function. Due to the presence of fever outside the hospital and persistent low‐grade fever upon admission, there was concern for pulmonary or urinary tract infection. Cefuroxime sodium was administered for anti‐infective treatment. Although the patient’s fever subsided after the above treatments, the headache symptoms persisted.

Due to objective constraints, FilmArray Meningitis/Encephalitis multiplex PCR testing and autoimmune encephalitis antibody testing were not performed. As the patient was initially misdiagnosed with viral encephalitis, acyclovir antiviral therapy was administered. Although this led to some relief of the headache, the symptoms persisted intermittently. Consequently, on the fourth day after admission, dexamethasone was added to the treatment regimen. The total course of dexamethasone treatment was 1 week, and the total course of acyclovir treatment was 2 weeks.

Following the comprehensive treatment mentioned above, the patient’s headache symptoms significantly improved. A repeat lumbar puncture showed a decrease in CSF protein levels compared to before. Ultimately, the headache symptoms nearly disappeared within 3 weeks. A telephone follow‐up after discharge indicated that the patient no longer experienced headache or fever symptoms.

## 3. Discussion

NIID manifests with a wide range of neurological symptoms that often overlap with other neurodegenerative disorders, including Alzheimer’s disease (AD), essential tremor (ET), Parkinson’s disease (PD), amyotrophic lateral sclerosis (ALS), and oculopharyngodistal myopathy (OPDM), posing substantial diagnostic challenges [6, 7]. Importantly, paroxysmal symptoms, including transient disturbances of consciousness, stroke‐like or encephalitis‐like episodes, generalized seizures, and migraine‐like attacks, represent a key feature that distinguishes NIID from other conditions [4, 8]. Previous studies indicate that approximately 66.8% of patients experience paroxysmal episodes during the disease course, often representing the predominant clinical manifestation [4], highlighting their critical role in improving diagnostic accuracy [9].

In this case, the patient had no prior history of neurological disorders and initially presented with acute headache and fever. CSF analysis revealed only mildly elevated protein levels, lacking specific etiological evidence, which initially led to a presumptive diagnosis of viral encephalitis. Notably, cranial MRI demonstrated abnormal signal distribution predominantly with restricted diffusion at the gray–white matter junction of both cerebral hemispheres. Compared with the CSF findings, this characteristic imaging feature provided an earlier indication of the potential for NIID. Subsequent pathological confirmation through skin and muscle biopsies, combined with genetic testing, ultimately established the diagnosis of NIID. Consistent with previously reported cases of NIID presenting with encephalitis‐like episodes [5, 10–13], this case suggests that acute inflammatory‐like manifestations may not represent an independent infectious process but rather constitute an early clinical phenotype of NIID, consistent with the encephalitis‐like symptoms observed among the paroxysmal manifestations of NIID described previously. By highlighting the diagnostic value of DWI features in specific clinical contexts, this study may enhance clinicians’ recognition of NIID presenting with encephalitis‐like episodes, support timely diagnostic decisions, and potentially facilitate early intervention to alleviate symptoms and improve patient quality of life. Furthermore, this case provides valuable reference for future research and the development of clinical diagnostic strategies for NIID.

Jun et al. systematically summarized the clinical features of NIID in a large‐scale study and found that a high proportion of patients presented with forgetfulness as their primary complaint, whereas paroxysmal symptoms were relatively rare [14]. The present case presented with acute‐onset headache and fever, which falls into the category of paroxysmal manifestations, differing from the typical clinical presentation of NIID reported by Jun et al. This discrepancy suggests that the clinical phenotype of NIID is heterogeneous, and some patients may present with acute paroxysmal symptoms as the initial manifestation rather than the typical chronic progressive cognitive dysfunction. Therefore, for patients presenting with unexplained acute encephalopathy‐like episodes in clinical practice, particularly those who do not respond to conventional treatment and exhibit other characteristic features of NIID (such as hyperintensity along the corticomedullary junction on DWI on brain MRI and peripheral neuropathy), NIID should be considered in the differential diagnosis even in the absence of typical complaints of forgetfulness.

The diagnosis of NIID typically requires a combination of diagnostic approaches, including CSF analysis, neuroimaging, skin biopsy, and histopathology. Regarding CSF analysis, previous studies have reported that protein and cell counts in the CSF of NIID patients may be mildly elevated or normal [14, 15]. Typical findings on cranial MRI in NIID include the following characteristic features: (i) DWI showing high signal at the cortical and medullary junctions, progressing from bilateral frontal lobes to bilateral parieto‐occipital lobes [16]. The linear high signal along the U‐fibers on DWI, coexisting with cognitive decline, is considered a characteristic feature of NIID [17]. Studies have shown a significant correlation between the severity of dementia and the white matter DWI high signal, unrelated to ventricular enlargement or brain atrophy [18]. (ii) As NIID progresses, T2/FLAIR high signals gradually affect the entire corpus callosum. It is speculated that the extent of abnormal signal in the corpus callosum is related to the progression of NIID [17].

Multiple studies have reported the presence of eosinophilic intranuclear inclusion bodies in skin biopsies of NIID patients. These inclusions are round, with a diameter of 1.5–10 μm, positive for ubiquitin and p62. Electron microscopy observations reveal that they are composed of fibrous materials lacking membrane structures [2, 14, 19, 20], making skin biopsy a diagnostic tool for NIID. However, the role of p62 in the pathogenesis of NIID remains unclear. As a ubiquitin‐binding protein, p62 plays a crucial role in various neurodegenerative diseases, but its significance in NIID is not yet fully understood [21]. Proteomic studies suggest that p62‐positive inclusions may increase the expression of nuclear proteins, including histones, tubulin proteins, and actin, and enhance the transcription of some hnRNAs inside the nucleus [22]. Further research is needed to explore the formation mechanism of these inclusions, their relationship with disease progression, and potential therapeutic targets.

Previous studies have linked GGC repeat expansions in the 5′ UTR of the NOTCH2NLC gene to the pathogenesis of NIID [3, 23]. Abnormal expansion of GGC trinucleotides in NIID provides a new molecular diagnostic approach for the disease [3, 24]. In this case, the diagnostic process involved a variety of examinations and assessments to rule out other neurological diseases and ultimately confirm the diagnosis of NIID. In addition to distinguishing it from viral encephalitis as mentioned earlier, it also needed to be differentiated from the following conditions: (i) Fragile X‐Associated Tremor/Ataxia Syndrome (FXTAS): This is a genetic disease that can also present with high signal intensity in the corticomedullary junction on MRI DWI sequences. However, its MRI features include symmetric high signal intensity in the middle cerebellar peduncles, global cerebellar atrophy, and lesions with high signal intensity in the splenium of the corpus callosum [25], which are different from NIID. It is worth noting that recent reports have demonstrated considerable overlap in MRI features between FXTAS and NIID, rendering imaging findings alone insufficient for reliable differentiation. Accordingly, confirmatory genetic testing remains essential. (ii) Mitochondrial Encephalomyopathy: The MRI findings in this disease typically involve high signal intensity on DWI in the temporoparietal cortical regions and subcortical white matter but generally do not affect the deep white matter [26], unlike NIID. (iii) Toxic Encephalopathy: Conditions like acute toluene poisoning may also present with high signal intensity in the subcortical and deep white matter [27], similar to NIID. However, since the patient had no history of toxic exposure, this condition was not considered.

Similar to other neurodegenerative diseases, there is currently no specific cure for NIID. Therefore, symptomatic treatment and rehabilitation care are the main therapeutic strategies. Research has reported that an NIID patient experienced symptom relief after receiving 3 days of methylprednisolone pulse therapy (500 mg/day) [5]. In another Japanese case report, intravenous immunoglobulin (IVIg) therapy improved the symptoms of a NIID patient [28]. In this case, the combination of acyclovir antiviral treatment and dexamethasone therapy significantly improved the patient’s headache and fever symptoms, suggesting that disease remission may be related to the neuroprotective effects of acyclovir [29] and dexamethasone [30]. Although studies have reported that patients with encephalitis‐like NIID respond well to glucocorticoid therapy [31], this case represents the first reported instance, to date, of significant symptom improvement in an NIID patient following treatment with acyclovir in combination with dexamethasone. However, the specific mechanisms involved need further elucidation. While there is currently no specific treatment for NIID, early intervention remains crucial. Timely intervention can delay disease progression, reduce the likelihood of deterioration, help prevent complications or slow their progression, and most importantly, potentially make treatments more effective.

Of note, in the existing literature, Tian et al. systematically summarized the clinical features of NIID and noted that an encephalitis‐like onset is relatively rare [4]. To date, only a few cases of NIID presenting with acute encephalitis‐like manifestations as the initial symptom have been reported [11, 31, 32]. In comparison with these cases, the clinical presentation of our patient was consistent, characterized by acute onset of fever and headache, elevated CSF protein, and eventual confirmation of the diagnosis by skin biopsy and genetic testing. However, unlike some previously reported cases [33], no epileptiform discharges were recorded on EEG in our patient. These similarities and differences suggest that the encephalitis‐like manifestations of NIID may exhibit certain clinical heterogeneity. Our case further expands the clinical spectrum of NIID and highlights that for patients with an unexplained acute encephalitis‐like episode, particularly those who do not respond to conventional anti‐infective therapy and exhibit albuminocytologic dissociation in the CSF, NIID should be considered in the differential diagnosis.

This case has several limitations. Firstly, only a single EEG was performed, and repeated evaluations during the clinical course were not available. The nonspecific EEG findings from a single recording are insufficient to exclude viral or autoimmune encephalitis, which to some extent limits the completeness of the diagnostic evaluation. Ideally, serial EEG monitoring should be performed in patients presenting with clinical symptoms suggestive of encephalitis. Secondly, due to objective constraints, FilmArray Meningitis/Encephalitis multiplex PCR testing and autoimmune encephalitis antibody testing were not performed, which to some extent limits the ability to definitively exclude infectious encephalitis. Nevertheless, given the absence of acute infection in CSF routine, biochemical, and serological findings, together with skin biopsy and genetic testing results both supporting a diagnosis of NIID, the likelihood of infectious or autoimmune encephalitis is considered low. Thirdly, the present case presented with headache as the initial symptom; however, the causal relationship between headache and NIID remains uncertain. Although common causes of headache were partially excluded through imaging, CSF, and serological examinations, and a final diagnosis of NIID was established, the possibility that the headache was attributable to other causes coexisting with NIID, or merely a clinical coincidence, cannot be ruled out. Therefore, this case does not directly prove that NIID was the cause of the headache. This limitation warrants further investigation in future studies with larger sample sizes. Lastly, in this case, only conventional HE staining was performed on the skin biopsy specimen, and p62 or ubiquitin immunohistochemical staining was not carried out. This limitation restricts, to some extent, the identification and confirmation of intranuclear inclusions. The ideal diagnostic workup should include p62 or ubiquitin immunohistochemistry to enhance diagnostic sensitivity. However, due to objective reasons, this examination was not completed, which constitutes a limitation of this case report.

In conclusion, we have reported a rare case of NIID. Through further research and clinical observations, we can better understand the pathogenesis of NIID, improve the accuracy of early diagnosis, and explore more effective treatment strategies, in order to provide patients with better medical services and care.

## Funding

This work was supported by the Zunyi City Science and Technology Innovation Talent Team Project (Zunyi Science Talent [2024] No. 6), Guizhou Provincial Health Commission Key Discipline Development Fund, and Department of Science and Technology of Guizhou Province ZSYS(2025)040.

## Consent

Written informed consent was obtained from the patient for publication of this case report and any accompanying images.

## Conflicts of Interest

The authors declare no conflicts of interest.

## Data Availability

Data sharing is not applicable to this article as no datasets were generated or analysed during the current study.
